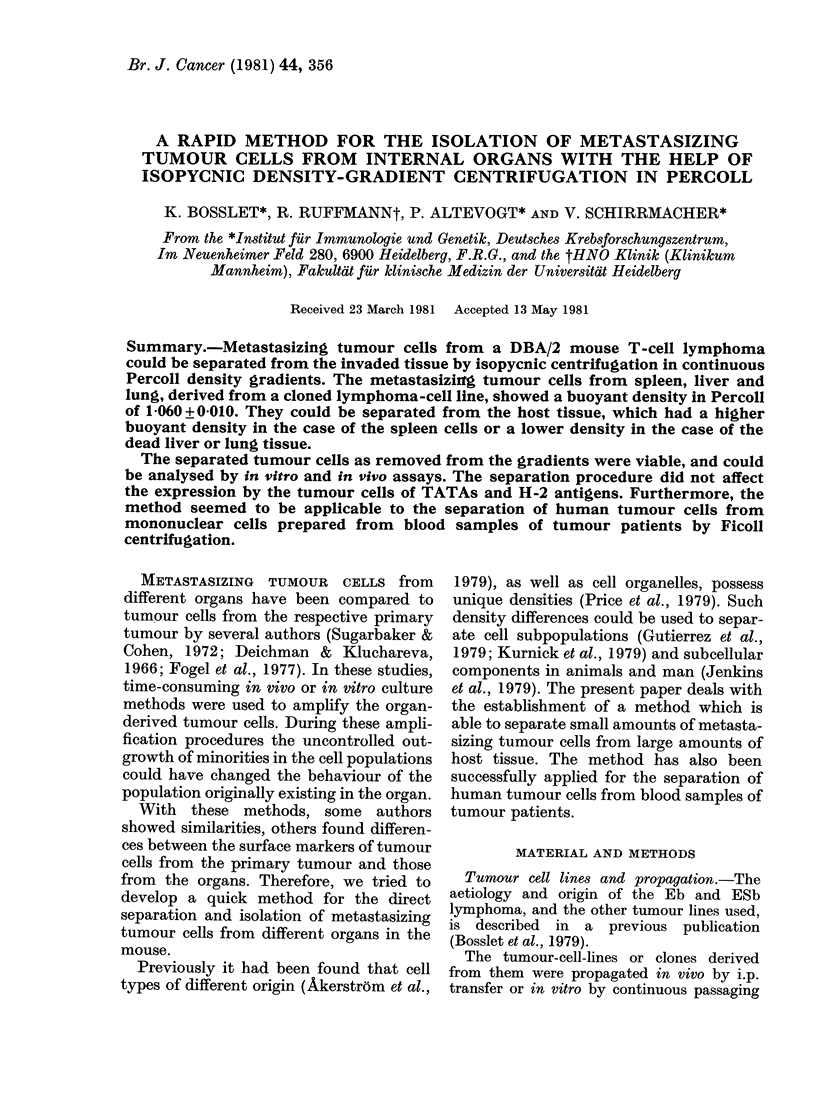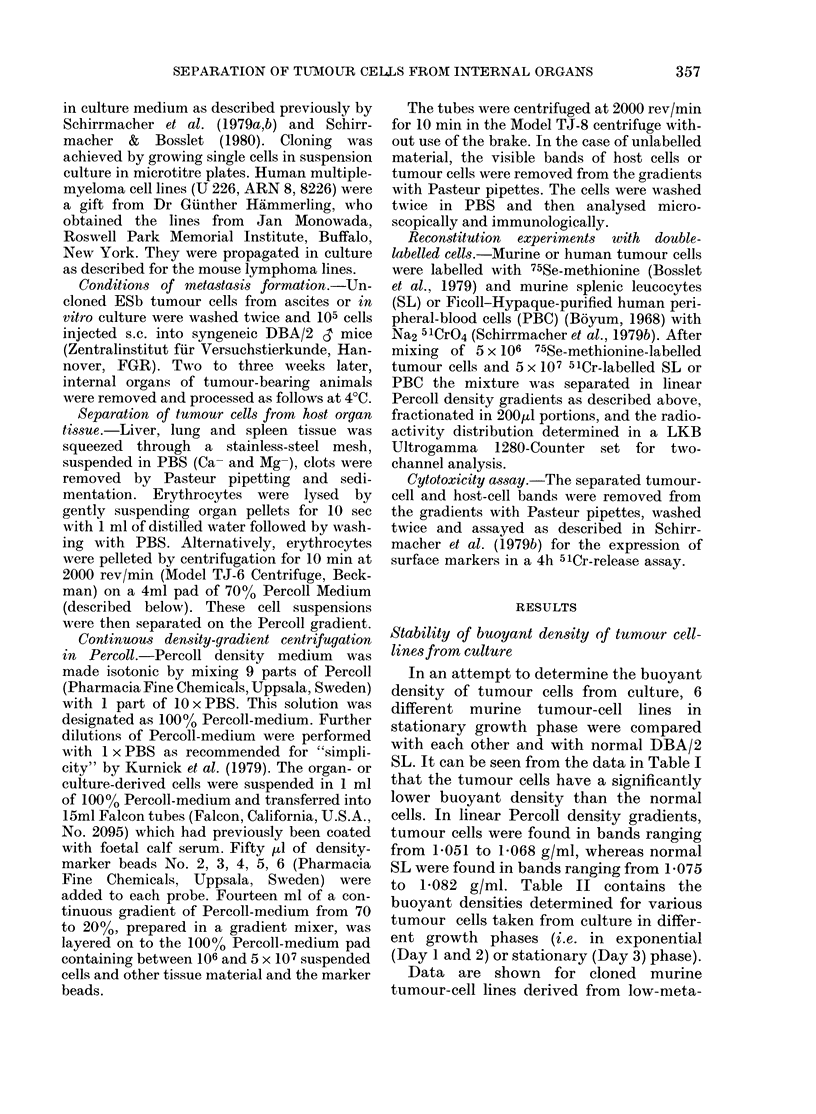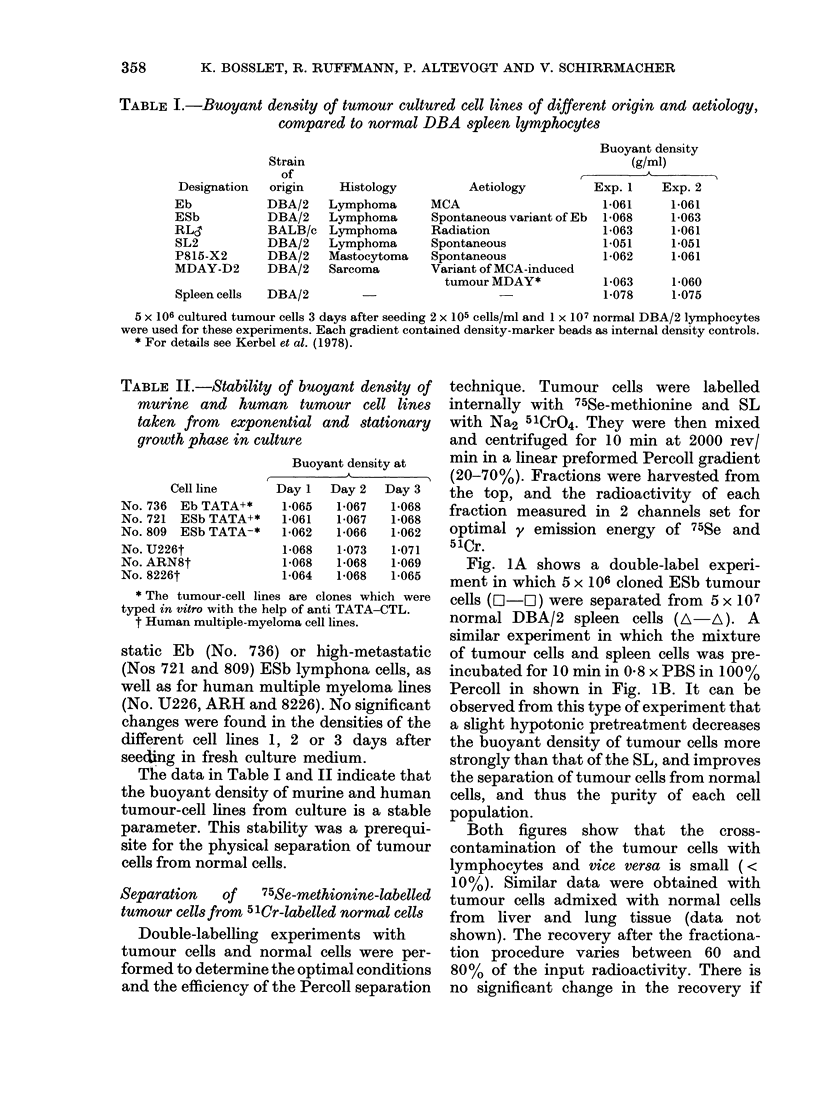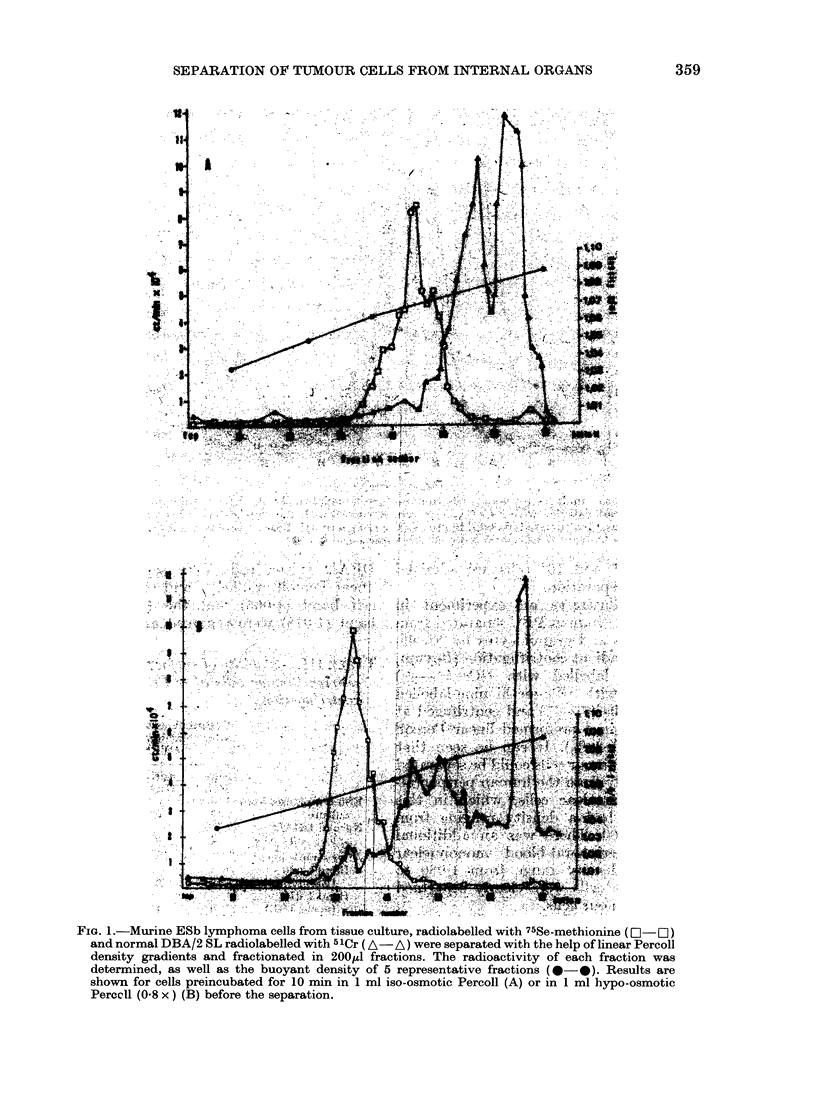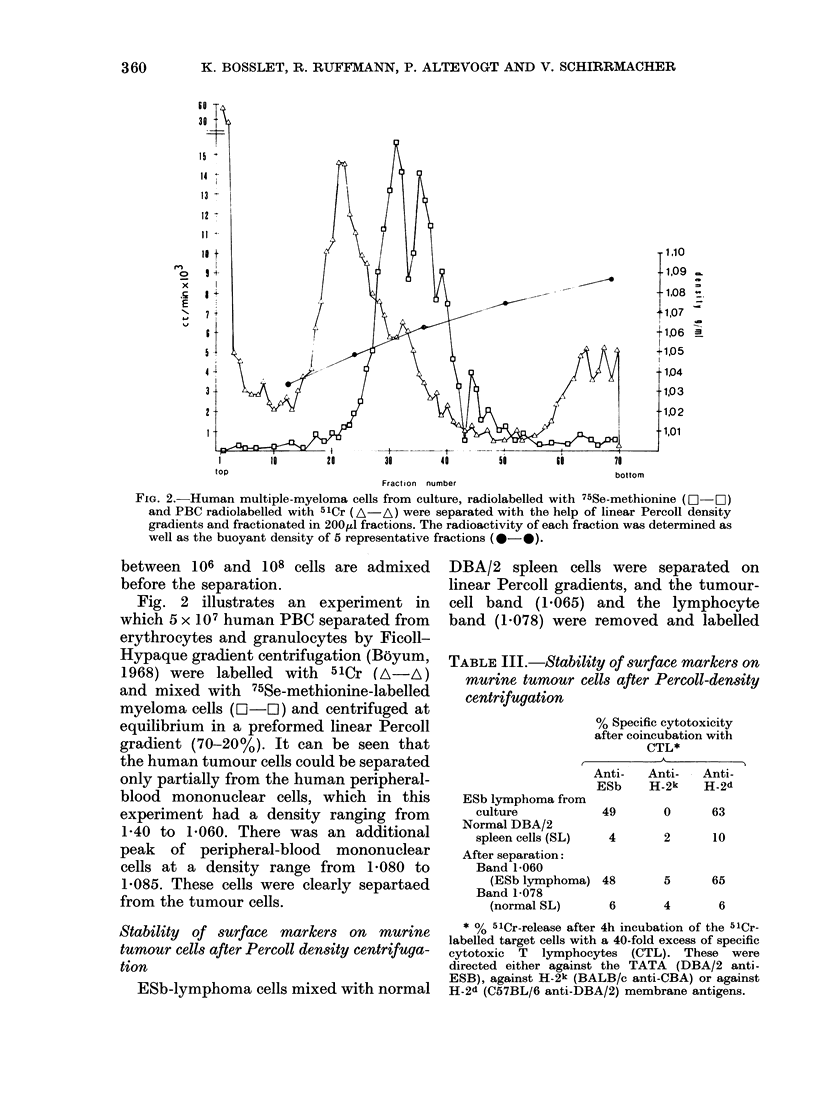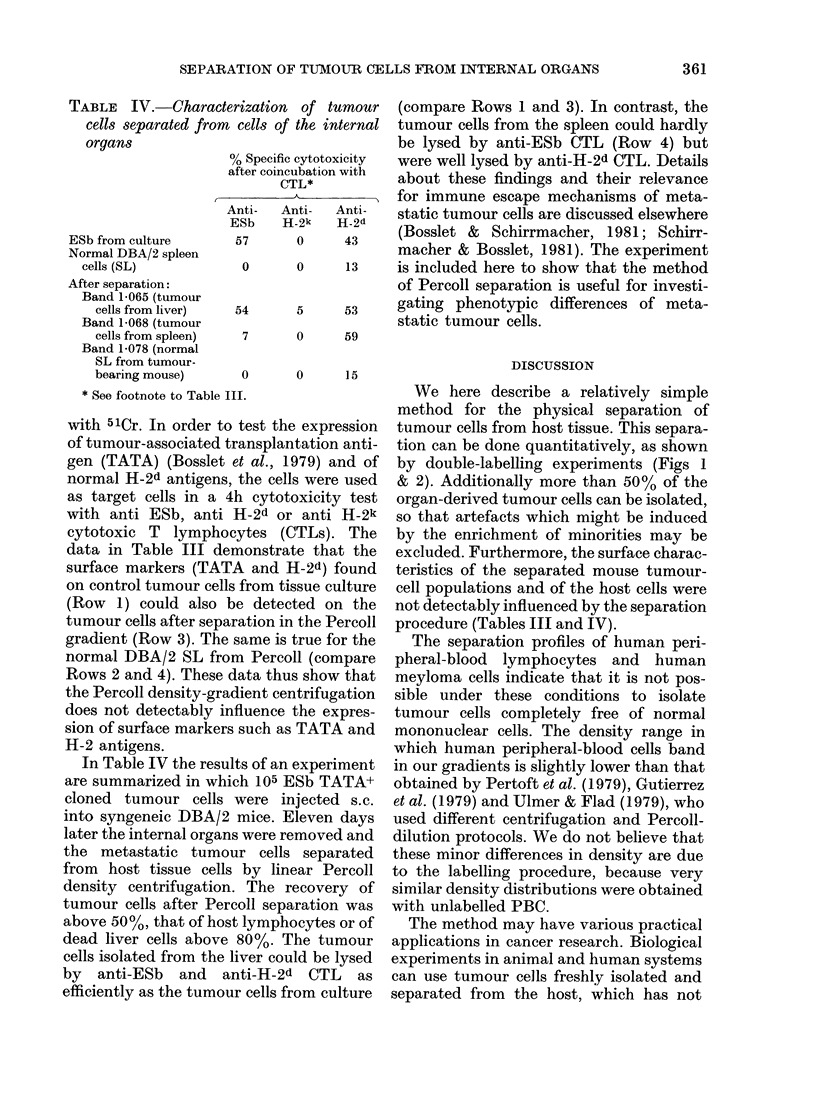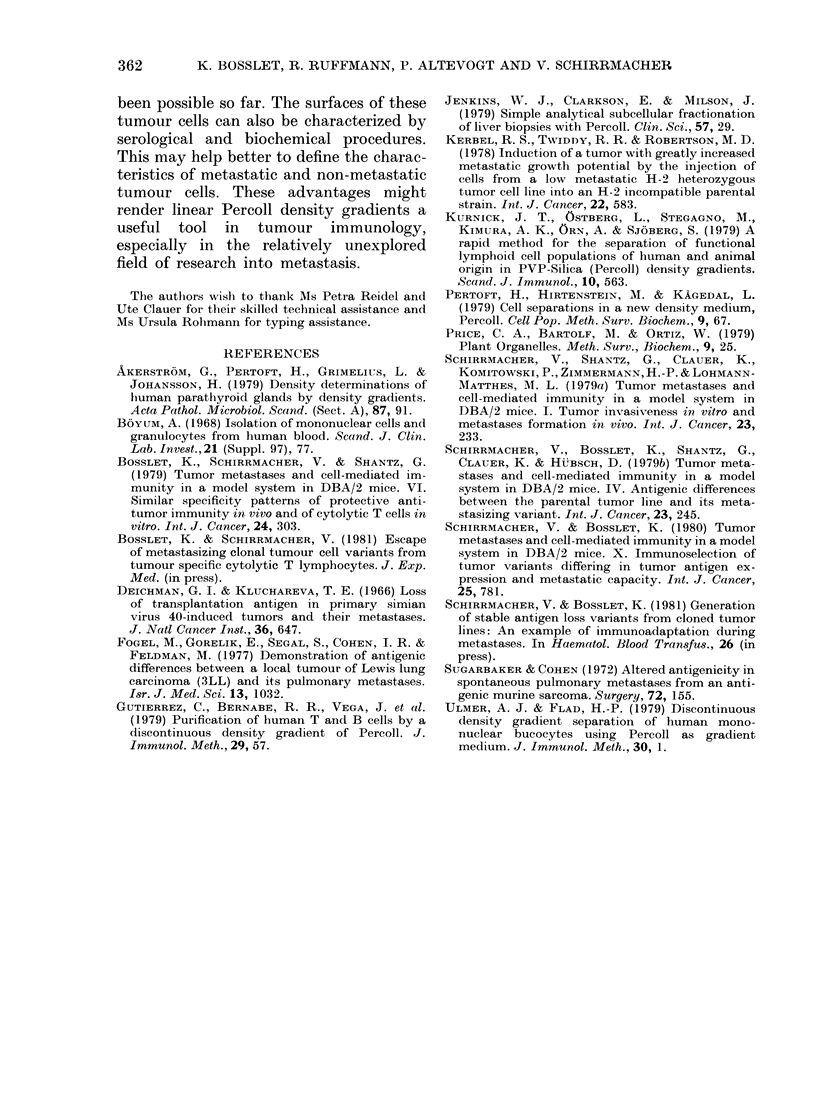# A rapid method for the isolation of metastasizing tumour cells from internal organs with the help of isopycnic density-gradient centrifugation in Percoll.

**DOI:** 10.1038/bjc.1981.192

**Published:** 1981-09

**Authors:** K. Bosslet, R. Ruffmann, P. Altevogt, V. Schirrmacher

## Abstract

Metastasizing tumour cells from a DBA/2 mouse T-cell lymphoma could be separated from the invaded tissue by isopycnic centrifugation in continuous Percoll density gradients. The metastasizing tumour cells from spleen, liver and lung, derived from a cloned lymphoma-cell line, showed a buoyant density in Percoll of 1.060 +/- 0.010. They could be separated from the host tissue, which had a higher buoyant density in the case of the spleen cells or a lower density in the case of the dead liver or lung tissue. The separated tumour cells as removed from the gradients were viable, and could be analysed by in vitro and in vivo assays. The separation procedure did not affect the expression by the tumour cells of TATAs and H-2 antigens. Furthermore, the method seemed to be applicable to the separation of human tumour cells from mononuclear cells prepared from blood samples of tumour patients by Ficoll centrifugation.


*Br. J. Cancer* (1981) **44**, 356

### A RAPID METHOD FOR THE ISOLATION OF METASTASIZING TUMOUR CELLS FROM INTERNAL ORGANS WITH THE HELP OF ISOPYCNIC DENSITY-GRADIENT CENTRIFUGATION IN PERCOLL

K. BOSSLET*, R. RUFFMANN†, P. ALTEVOGT* AND V. SCHIRRMACHER*

*From the *Institut für Immunologie und Genetik, Deutsches Krebsforschungszentrum, Im Neuenheimer Feld* 280, 6900 *Heidelberg, F.R.G., and the †HNO Klinik (Klinikum Mannheim), Fakultät für klinische Medizin der Universität Heidelberg*

Received 23 March 1981 Accepted 13 May 1981

**Summary.—Metastasizing tumour cells from a DBA/2 mouse T-cell lymphoma could be separated from the invaded tissue by isopycnic centrifugation in continuous Percoll density gradients. The metastasizing tumour cells from spleen, liver and lung, derived from a cloned lymphoma-cell line, showed a buoyant density in Percoll of 1·060 ± 0·010. They could be separated from the host tissue, which had a higher buoyant density in the case of the spleen cells or a lower density in the case of the dead liver or lung tissue.**

**The separated tumour cells as removed from the gradients were viable, and could be analysed by *in vitro* and *in vivo* assays. The separation procedure did not affect the expression by the tumour cells of TATAs and H-2 antigens. Furthermore, the method seemed to be applicable to the separation of human tumour cells from mononuclear cells prepared from blood samples of tumour patients by Ficoll centrifugation.**

METASTASIZING TUMOUR CELLS from different organs have been compared to tumour cells from the respective primary tumour by several authors (Sugarbaker & Cohen, 1972; Deichman & Kluchareva, 1966; Fogel *et al.*, 1977). In these studies, time-consuming *in vivo* or *in vitro* culture methods were used to amplify the organ-derived tumour cells. During these amplification procedures the uncontrolled outgrowth of minorities in the cell populations could have changed the behaviour of the population originally existing in the organ.

With these methods, some authors showed similarities, others found differences between the surface markers of tumour cells from the primary tumour and those from the organs. Therefore, we tried to develop a quick method for the direct separation and isolation of metastasizing tumour cells from different organs in the mouse.

Previously it had been found that cell types of different origin (Åkerström *et al.*, 1979), as well as cell organelles, possess unique densities (Price *et al.*, 1979). Such density differences could be used to separate cell subpopulations (Gutierrez *et al.*, 1979; Kurnick *et al.*, 1979) and subcellular components in animals and man (Jenkins *et al.*, 1979). The present paper deals with the establishment of a method which is able to separate small amounts of metastasizing tumour cells from large amounts of host tissue. The method has also been successfully applied for the separation of human tumour cells from blood samples of tumour patients.

### MATERIAL AND METHODS

*Tumour cell lines and propagation.*—The aetiology and origin of the Eb and ESb lymphoma, and the other tumour lines used, is described in a previous publication (Bosslet *et al.*, 1979).

The tumour-cell-lines or clones derived from them were propagated *in vivo* by i.p. transfer or *in vitro* by continuous passaging in culture medium as described previously by Schirrmacher *et al.* (1979*a,b*) and Schirrmacher & Bosslet (1980). Cloning was achieved by growing single cells in suspension culture in microtitre plates. Human multiple-myeloma cell lines (U 226, ARN 8, 8226) were a gift from Dr Günther Hämmerling, who obtained the lines from Jan Monowada, Roswell Park Memorial Institute, Buffalo, New York. They were propagated in culture as described for the mouse lymphoma lines.

*Conditions of metastasis formation.*—Uncloned ESb tumour cells from ascites or *in vitro* culture were washed twice and $10^5$ cells injected s.c. into syngeneic DBA/2 ♂ mice (Zentralinstitut für Versuchstierkunde, Hannover, FGR). Two to three weeks later, internal organs of tumour-bearing animals were removed and processed as follows at 4°C.

*Separation of tumour cells from host organ tissue.*—Liver, lung and spleen tissue was squeezed through a stainless-steel mesh, suspended in PBS ($Ca^-$ and $Mg^-$), clots were removed by Pasteur pipetting and sedimentation. Erythrocytes were lysed by gently suspending organ pellets for 10 sec with 1 ml of distilled water followed by washing with PBS. Alternatively, erythrocytes were pelleted by centrifugation for 10 min at 2000 rev/min (Model TJ-6 Centrifuge, Beckman) on a 4ml pad of 70% Percoll Medium (described below). These cell suspensions were then separated on the Percoll gradient.

*Continuous density-gradient centrifugation in Percoll.*—Percoll density medium was made isotonic by mixing 9 parts of Percoll (Pharmacia Fine Chemicals, Uppsala, Sweden) with 1 part of 10 × PBS. This solution was designated as 100% Percoll-medium. Further dilutions of Percoll-medium were performed with 1 × PBS as recommended for "simplicity" by Kurnick *et al.* (1979). The organ- or culture-derived cells were suspended in 1 ml of 100% Percoll-medium and transferred into 15ml Falcon tubes (Falcon, California, U.S.A., No. 2095) which had previously been coated with foetal calf serum. Fifty μl of density-marker beads No. 2, 3, 4, 5, 6 (Pharmacia Fine Chemicals, Uppsala, Sweden) were added to each probe. Fourteen ml of a continuous gradient of Percoll-medium from 70 to 20%, prepared in a gradient mixer, was layered on to the 100% Percoll-medium pad containing between $10^6$ and $5 \times 10^7$ suspended cells and other tissue material and the marker beads.

The tubes were centrifuged at 2000 rev/min for 10 min in the Model TJ-8 centrifuge without use of the brake. In the case of unlabelled material, the visible bands of host cells or tumour cells were removed from the gradients with Pasteur pipettes. The cells were washed twice in PBS and then analysed microscopically and immunologically.

*Reconstitution experiments with double-labelled cells.*—Murine or human tumour cells were labelled with $^{75}$Se-methionine (Bosslet *et al.*, 1979) and murine splenic leucocytes (SL) or Ficoll–Hypaque-purified human peripheral-blood cells (PBC) (Böyum, 1968) with $Na_2{}^{51}CrO_4$ (Schirrmacher *et al.*, 1979*b*). After mixing of $5 \times 10^6$ $^{75}$Se-methionine-labelled tumour cells and $5 \times 10^7$ $^{51}$Cr-labelled SL or PBC the mixture was separated in linear Percoll density gradients as described above, fractionated in 200μl portions, and the radioactivity distribution determined in a LKB Ultrogamma 1280-Counter set for two-channel analysis.

*Cytotoxicity assay.*—The separated tumour-cell and host-cell bands were removed from the gradients with Pasteur pipettes, washed twice and assayed as described in Schirrmacher *et al.* (1979*b*) for the expression of surface markers in a 4h $^{51}$Cr-release assay.

### RESULTS

### *Stability of buoyant density of tumour cell-lines from culture*

In an attempt to determine the buoyant density of tumour cells from culture, 6 different murine tumour-cell lines in stationary growth phase were compared with each other and with normal DBA/2 SL. It can be seen from the data in Table I that the tumour cells have a significantly lower buoyant density than the normal cells. In linear Percoll density gradients, tumour cells were found in bands ranging from 1·051 to 1·068 g/ml, whereas normal SL were found in bands ranging from 1·075 to 1·082 g/ml. Table II contains the buoyant densities determined for various tumour cells taken from culture in different growth phases (*i.e.* in exponential (Day 1 and 2) or stationary (Day 3) phase).

Data are shown for cloned murine tumour-cell lines derived from low-meta-

TABLE I.—*Buoyant density of tumour cultured cell lines of different origin and aetiology, compared to normal DBA spleen lymphocytes*

| Designation | Strain of origin | Histology | Aetiology | Buoyant density (g/ml) Exp. 1 | Exp. 2 |
|---|---|---|---|---|---|
| Eb | DBA/2 | Lymphoma | MCA | 1·061 | 1·061 |
| ESb | DBA/2 | Lymphoma | Spontaneous variant of Eb | 1·068 | 1·063 |
| RL♂ | BALB/c | Lymphoma | Radiation | 1·063 | 1·061 |
| SL2 | DBA/2 | Lymphoma | Spontaneous | 1·051 | 1·051 |
| P815-X2 | DBA/2 | Mastocytoma | Spontaneous | 1·062 | 1·061 |
| MDAY-D2 | DBA/2 | Sarcoma | Variant of MCA-induced tumour MDAY* | 1·063 | 1·060 |
| Spleen cells | DBA/2 | — | — | 1·078 | 1·075 |

$5 \times 10^6$ cultured tumour cells 3 days after seeding $2 \times 10^5$ cells/ml and $1 \times 10^7$ normal DBA/2 lymphocytes were used for these experiments. Each gradient contained density-marker beads as internal density controls.
* For details see Kerbel *et al.* (1978).

TABLE II.—*Stability of buoyant density of murine and human tumour cell lines taken from exponential and stationary growth phase in culture*

| Cell line | | Buoyant density at Day 1 | Day 2 | Day 3 |
|---|---|---|---|---|
| No. 736 | Eb TATA$^+$* | 1·065 | 1·067 | 1·068 |
| No. 721 | ESb TATA$^+$* | 1·061 | 1·067 | 1·068 |
| No. 809 | ESb TATA$^-$* | 1·062 | 1·066 | 1·062 |
| No. U226† | | 1·068 | 1·073 | 1·071 |
| No. ARN8† | | 1·068 | 1·068 | 1·069 |
| No. 8226† | | 1·064 | 1·068 | 1·065 |

* The tumour-cell lines are clones which were typed *in vitro* with the help of anti TATA–CTL.
† Human multiple-myeloma cell lines.

static Eb (No. 736) or high-metastatic (Nos 721 and 809) ESb lymphona cells, as well as for human multiple myeloma lines (No. U226, ARH and 8226). No significant changes were found in the densities of the different cell lines 1, 2 or 3 days after seeding in fresh culture medium.

The data in Table I and II indicate that the buoyant density of murine and human tumour-cell lines from culture is a stable parameter. This stability was a prerequisite for the physical separation of tumour cells from normal cells.

*Separation of $^{75}$Se-methionine-labelled tumour cells from $^{51}$Cr-labelled normal cells*

Double-labelling experiments with tumour cells and normal cells were performed to determine the optimal conditions and the efficiency of the Percoll separation technique. Tumour cells were labelled internally with $^{75}$Se-methionine and SL with $Na_2\,{}^{51}CrO_4$. They were then mixed and centrifuged for 10 min at 2000 rev/min in a linear preformed Percoll gradient (20–70%). Fractions were harvested from the top, and the radioactivity of each fraction measured in 2 channels set for optimal $\gamma$ emission energy of $^{75}$Se and $^{51}$Cr.

Fig. 1A shows a double-label experiment in which $5 \times 10^6$ cloned ESb tumour cells (□—□) were separated from $5 \times 10^7$ normal DBA/2 spleen cells (△—△). A similar experiment in which the mixture of tumour cells and spleen cells was preincubated for 10 min in $0{\cdot}8 \times$ PBS in 100% Percoll in shown in Fig. 1B. It can be observed from this type of experiment that a slight hypotonic pretreatment decreases the buoyant density of tumour cells more strongly than that of the SL, and improves the separation of tumour cells from normal cells, and thus the purity of each cell population.

Both figures show that the cross-contamination of the tumour cells with lymphocytes and *vice versa* is small ($<$ 10%). Similar data were obtained with tumour cells admixed with normal cells from liver and lung tissue (data not shown). The recovery after the fractionation procedure varies between 60 and 80% of the input radioactivity. There is no significant change in the recovery if

FIG. 1.—Murine ESb lymphoma cells from tissue culture, radiolabelled with $^{75}$Se-methionine (□—□) and normal DBA/2 SL radiolabelled with $^{51}$Cr (△—△) were separated with the help of linear Percoll density gradients and fractionated in 200$\mu$l fractions. The radioactivity of each fraction was determined, as well as the buoyant density of 5 representative fractions (●—●). Results are shown for cells preincubated for 10 min in 1 ml iso-osmotic Percoll (A) or in 1 ml hypo-osmotic Percoll (0·8 ×) (B) before the separation.

FIG. 2.—Human multiple-myeloma cells from culture, radiolabelled with $^{75}$Se-methionine (□—□) and PBC radiolabelled with $^{51}$Cr (△—△) were separated with the help of linear Percoll density gradients and fractionated in 200$\mu$l fractions. The radioactivity of each fraction was determined as well as the buoyant density of 5 representative fractions (●—●).

between $10^6$ and $10^8$ cells are admixed before the separation.

Fig. 2 illustrates an experiment in which $5 \times 10^7$ human PBC separated from erythrocytes and granulocytes by Ficoll–Hypaque gradient centrifugation (Böyum, 1968) were labelled with $^{51}$Cr (△—△) and mixed with $^{75}$Se-methionine-labelled myeloma cells (□—□) and centrifuged at equilibrium in a preformed linear Percoll gradient (70–20%). It can be seen that the human tumour cells could be separated only partially from the human peripheral-blood mononuclear cells, which in this experiment had a density ranging from 1·40 to 1·060. There was an additional peak of peripheral-blood mononuclear cells at a density range from 1·080 to 1·085. These cells were clearly separtaed from the tumour cells.

### *Stability of surface markers on murine tumour cells after Percoll density centrifugation*

ESb-lymphoma cells mixed with normal DBA/2 spleen cells were separated on linear Percoll gradients, and the tumour-cell band (1·065) and the lymphocyte band (1·078) were removed and labelled

TABLE III.—*Stability of surface markers on murine tumour cells after Percoll-density centrifugation*

| | % Specific cytotoxicity after coincubation with CTL* | | |
|---|---|---|---|
| | Anti-ESb | Anti-H-2$^k$ | Anti-H-2$^d$ |
| ESb lymphoma from culture | 49 | 0 | 63 |
| Normal DBA/2 spleen cells (SL) | 4 | 2 | 10 |
| After separation: | | | |
| Band 1·060 (ESb lymphoma) | 48 | 5 | 65 |
| Band 1·078 (normal SL) | 6 | 4 | 6 |

* % $^{51}$Cr-release after 4h incubation of the $^{51}$Cr-labelled target cells with a 40-fold excess of specific cytotoxic T lymphocytes (CTL). These were directed either against the TATA (DBA/2 anti-ESB), against H-2$^k$ (BALB/c anti-CBA) or against H-2$^d$ (C57BL/6 anti-DBA/2) membrane antigens.

TABLE IV.—*Characterization of tumour cells separated from cells of the internal organs*

| | % Specific cytotoxicity after coincubation with CTL* | | |
|---|---|---|---|
| | Anti-ESb | Anti-H-$2^k$ | Anti-H-$2^d$ |
| ESb from culture | 57 | 0 | 43 |
| Normal DBA/2 spleen cells (SL) | 0 | 0 | 13 |
| After separation: | | | |
| Band 1·065 (tumour cells from liver) | 54 | 5 | 53 |
| Band 1·068 (tumour cells from spleen) | 7 | 0 | 59 |
| Band 1·078 (normal SL from tumour-bearing mouse) | 0 | 0 | 15 |

* See footnote to Table III.

with $^{51}$Cr. In order to test the expression of tumour-associated transplantation antigen (TATA) (Bosslet *et al.*, 1979) and of normal H-$2^d$ antigens, the cells were used as target cells in a 4h cytotoxicity test with anti ESb, anti H-$2^d$ or anti H-$2^k$ cytotoxic T lymphocytes (CTLs). The data in Table III demonstrate that the surface markers (TATA and H-$2^d$) found on control tumour cells from tissue culture (Row 1) could also be detected on the tumour cells after separation in the Percoll gradient (Row 3). The same is true for the normal DBA/2 SL from Percoll (compare Rows 2 and 4). These data thus show that the Percoll density-gradient centrifugation does not detectably influence the expression of surface markers such as TATA and H-2 antigens.

In Table IV the results of an experiment are summarized in which $10^5$ ESb TATA$^+$ cloned tumour cells were injected s.c. into syngeneic DBA/2 mice. Eleven days later the internal organs were removed and the metastatic tumour cells separated from host tissue cells by linear Percoll density centrifugation. The recovery of tumour cells after Percoll separation was above 50%, that of host lymphocytes or of dead liver cells above 80%. The tumour cells isolated from the liver could be lysed by anti-ESb and anti-H-$2^d$ CTL as efficiently as the tumour cells from culture (compare Rows 1 and 3). In contrast, the tumour cells from the spleen could hardly be lysed by anti-ESb CTL (Row 4) but were well lysed by anti-H-$2^d$ CTL. Details about these findings and their relevance for immune escape mechanisms of metastatic tumour cells are discussed elsewhere (Bosslet & Schirrmacher, 1981; Schirrmacher & Bosslet, 1981). The experiment is included here to show that the method of Percoll separation is useful for investigating phenotypic differences of metastatic tumour cells.

### DISCUSSION

We here describe a relatively simple method for the physical separation of tumour cells from host tissue. This separation can be done quantitatively, as shown by double-labelling experiments (Figs 1 & 2). Additionally more than 50% of the organ-derived tumour cells can be isolated, so that artefacts which might be induced by the enrichment of minorities may be excluded. Furthermore, the surface characteristics of the separated mouse tumour-cell populations and of the host cells were not detectably influenced by the separation procedure (Tables III and IV).

The separation profiles of human peripheral-blood lymphocytes and human meyloma cells indicate that it is not possible under these conditions to isolate tumour cells completely free of normal mononuclear cells. The density range in which human peripheral-blood cells band in our gradients is slightly lower than that obtained by Pertoft *et al.* (1979), Gutierrez *et al.* (1979) and Ulmer & Flad (1979), who used different centrifugation and Percoll-dilution protocols. We do not believe that these minor differences in density are due to the labelling procedure, because very similar density distributions were obtained with unlabelled PBC.

The method may have various practical applications in cancer research. Biological experiments in animal and human systems can use tumour cells freshly isolated and separated from the host, which has not been possible so far. The surfaces of these tumour cells can also be characterized by serological and biochemical procedures. This may help better to define the characteristics of metastatic and non-metastatic tumour cells. These advantages might render linear Percoll density gradients a useful tool in tumour immunology, especially in the relatively unexplored field of research into metastasis.

The authors wish to thank Ms Petra Reidel and Ute Clauer for their skilled technical assistance and Ms Ursula Rohmann for typing assistance.

### REFERENCES

ÅKERSTRÖM, G., PERTOFT, H., GRIMELIUS, L. & JOHANSSON, H. (1979) Density determinations of human parathyroid glands by density gradients. *Acta Pathol. Microbiol. Scand.* (Sect. A), **87**, 91.

BÖYUM, A. (1968) Isolation of mononuclear cells and granulocytes from human blood. *Scand. J. Clin. Lab. Invest.*, **21** (Suppl. 97), 77.

BOSSLET, K., SCHIRRMACHER, V. & SHANTZ, G. (1979) Tumor metastases and cell-mediated immunity in a model system in DBA/2 mice. VI. Similar specificity patterns of protective antitumor immunity *in vivo* and of cytolytic T cells *in vitro*. *Int. J. Cancer*, **24**, 303.

BOSSLET, K. & SCHIRRMACHER, V. (1981) Escape of metastasizing clonal tumour cell variants from tumour specific cytolytic T lymphocytes. *J. Exp. Med.* (in press).

DEICHMAN, G. I. & KLUCHAREVA, T. E. (1966) Loss of transplantation antigen in primary simian virus 40-induced tumors and their metastases. *J. Natl Cancer Inst.*, **36**, 647.

FOGEL, M., GORELIK, E., SEGAL, S., COHEN, I. R. & FELDMAN, M. (1977) Demonstration of antigenic differences between a local tumour of Lewis lung carcinoma (3LL) and its pulmonary metastases. *Isr. J. Med. Sci.* **13**, 1032.

GUTIERREZ, C., BERNABE, R. R., VEGA, J. *et al.* (1979) Purification of human T and B cells by a discontinuous density gradient of Percoll. *J. Immunol. Meth.*, **29**, 57.

JENKINS, W. J., CLARKSON, E. & MILSON, J. (1979) Simple analytical subcellular fractionation of liver biopsies with Percoll. *Clin. Sci.*, **57**, 29.

KERBEL, R. S., TWIDDY, R. R. & ROBERTSON, M. D. (1978) Induction of a tumor with greatly increased metastatic growth potential by the injection of cells from a low metastatic H-2 heterozygous tumor cell line into an H-2 incompatible parental strain. *Int. J. Cancer*, **22**, 583.

KURNICK, J. T., ÖSTBERG, L., STEGAGNO, M., KIMURA, A. K., ÖRN, A. & SJÖBERG, S. (1979) A rapid method for the separation of functional lymphoid cell populations of human and animal origin in PVP-Silica (Percoll) density gradients. *Scand. J. Immunol.*, **10**, 563.

PERTOFT, H., HIRTENSTEIN, M. & KÅGEDAL, L. (1979) Cell separations in a new density medium, Percoll. *Cell Pop. Meth. Surv. Biochem.*, **9**, 67.

PRICE, C. A., BARTOLF, M. & ORTIZ, W. (1979) Plant Organelles. *Meth. Surv., Biochem.*, **9**, 25.

SCHIRRMACHER, V., SHANTZ, G., CLAUER, K., KOMITOWSKI, P., ZIMMERMANN, H.-P. & LOHMANN-MATTHES, M. L. (1979*a*) Tumor metastases and cell-mediated immunity in a model system in DBA/2 mice. I. Tumor invasiveness *in vitro* and metastases formation *in vivo*. *Int. J. Cancer*, **23**, 233.

SCHIRRMACHER, V., BOSSLET, K., SHANTZ, G., CLAUER, K. & HÜBSCH, D. (1979*b*) Tumor metastases and cell-mediated immunity in a model system in DBA/2 mice. IV. Antigenic differences between the parental tumor line and its metastasizing variant. *Int. J. Cancer*, **23**, 245.

SCHIRRMACHER, V. & BOSSLET, K. (1980) Tumor metastases and cell-mediated immunity in a model system in DBA/2 mice. X. Immunoselection of tumor variants differing in tumor antigen expression and metastatic capacity. *Int. J. Cancer*, **25**, 781.

SCHIRRMACHER, V. & BOSSLET, K. (1981) Generation of stable antigen loss variants from cloned tumor lines: An example of immunoadaptation during metastases. In *Haematol. Blood Transfus.*, **26** (in press).

SUGARBAKER & COHEN (1972) Altered antigenicity in spontaneous pulmonary metastases from an antigenic murine sarcoma. *Surgery*, **72**, 155.

ULMER, A. J. & FLAD, H.-P. (1979) Discontinuous density gradient separation of human mononuclear bucocytes using Percoll as gradient medium. *J. Immunol. Meth.*, **30**, 1.